# Considering sex and gender in Epidemiology: a challenge beyond terminology. From conceptual analysis to methodological strategies

**DOI:** 10.1186/s13293-022-00430-6

**Published:** 2022-05-12

**Authors:** Hélène Colineaux, Alexandra Soulier, Benoit Lepage, Michelle Kelly-Irving

**Affiliations:** 1 EQUITY Team,, CERPOP, INSERM, Batiment E, 1er Etage, 37 Allées Jules Guesde, 31062 Toulouse, France; 2 IHPST UMR 8590, CNRS, 13 rue du Four, 75006 Paris, France; 3 Epidemiology Department, CHU Toulouse, 37 Allées Jules Guesde, 31062 Toulouse, France; 4 Biostatistic Department, Toulouse III University, 37 Allées Jules Guesde, 31062 Toulouse, France

**Keywords:** Gender, Sex, Epidemiology, Health inequality, Embodiment, Quantitative methods, Mediation analysis, Causal analysis, Pathways, Interaction

## Abstract

**Background:**

Epidemiologists need tools to measure effects of *gender*, a complex concept originating in the social sciences which is not easily operationalized in the discipline. Our aim is to clarify useful concepts, measures, paths, effects, and analytical strategies to explore mechanisms of health difference between men and women.

**Methods:**

We reviewed concepts to clarify their definitions and limitations for their translation into usable measures in Epidemiology. Then we conducted methodological research using a causal framework to propose methodologically appropriate strategies for measuring sex and gender effects in health.

**Results:**

(1) Concepts and measures. We define *gender* as a set of norms prescribed to individuals according to their attributed-at-birth sex. G*ender pressure* creates a systemic gap, at population level, in behaviors, activities, experiences, etc., between men and women. A pragmatic individual measure of gender would correspond to the level at which an individual complies with a set of elements constituting femininity or masculinity in a given population, place and time. (2) Main analytical strategy. Defining and measuring gender are not sufficient to distinguish the effects of sex and gender on a health outcome. We should also think in terms of mechanisms, i.e., how the variables are linked together, to define appropriate analytical strategies. A causal framework can help us to conceptualize “sex” as a “parent” of a gender or gendered variable. This implies that we cannot interpret sex effects as sexed mechanisms, and that we can explore gendered mechanisms of sex-differences by mediation analyses. (3) Alternative strategy. Gender could also be directly examined as a mechanism, rather than through a variable representing its realization in the individual, by approaching it as an interaction between sex and social environment.

**Conclusions:**

Both analytical strategies have limitations relative to the impossibility of reducing a complex concept to a single or a few measures, and of capturing the entire effect of the phenomenon of gender. However, these strategies could lead to more accurate analyses of the mechanisms underlying health differences between men and women.

## Highlights

- A pragmatic individual measure of individual gender would correspond to the level at which an individual complies with a set of elements constituting femininity or masculinity in a given population, place and time
- Comparing outcomes by sex and gender is not sufficient, and even misleading, to understand the mechanisms underlying biological and health differences between men and women
- Causal analysis framework is a powerful tool for refining hypotheses and identifying the effects that can and should be estimated to meet the objectives
- Gender could also be measured at the populational-level as an interaction between sex and environment, which would be more compatible with the sociological concept of gender

## Introduction

For more than a decade, scholars have been working to clarify sex and gender terms for biomedical research and have emphasized that they should not be confused [1–5]. However, although the importance of taking these factors into account in epidemiology^1^ has been repeatedly emphasized [6, 7], their true integration into practice remains marginal and often approximate [8]. This observation could be due to a poor understanding of the definitions by health researchers, since these terms are often used as synonyms, or interchangeably in scientific papers [3, 9–12]. We hypothesize that these persistent difficulties are partly based on lack of adaptation of these concepts imported from disciplines that do not have same challenges, paradigms and methods as epidemiology. We aim to transpose considerations about sex and gender developed in social sciences and humanities and propose a pragmatic operationalization of them, so that they can be better implemented in our field.

In the medical sciences, definitions of the terms "sex" and "gender" consensually follow the classic definitions given by sociologist Oakley in the 1970s [13]: sex refers to biological differences between women and men [1, 2] and gender refers to observed, experienced, prescribed or favored social differences, based on attributed-at-birth sex [1–4]. The concept of "sex" can be more precisely understood as a social construct [14, 15], premised upon a set of biological characteristics of different natures (genes, hormones, anatomy, etc.), directly or indirectly in connection with reproductive function, and on average strongly correlated with each other within each sex category [2, 10]. About the concept of “gender”, the minimalistic definition of "*social differences between men and women*" [1] hides the complexity of this concept. Gender is indeed a complex phenomenon which is continuous (femininity ↔ masculinity) but strongly premised upon the binary classification of sex, multidimensional (traits, norms, stereotypes, roles, responsibilities, activities, etc.), multi-level (experienced by individuals and prescribed by society, at different structural levels, and possibly heterogeneously), intersecting (with age, ethnicity, class, etc.), highly contextual, evolving over the life course, and across generations, and highly diffuse (in society, family, work… in relations, in expectations, in perceptions, in actions, etc.).

Epidemiology sometimes investigates differences in health between men and women. As such, sex can be regarded as the *exposure* of interest. In this case, the central question posed is: why are there differences in health between men and women? The phenomena at stake in these differences can be social and/or biological and therefore involve the concepts of sex and gender in a deeply intertwined way. For example, why are little boys more likely to have allergic diseases than little girls? Are there biological predispositions related to biological sex or is it the socially defined gendered exposures that explains it? Of course, the nature of the mechanisms sometimes seems obvious. Especially if the outcome is social: it seems obvious that the difference in wages between men and women is not directly determined by their respective reproductive physiology. This is less obvious for "biological" differences such as cholesterol levels, cortisol levels, allostatic load, etc. Some researchers implicitly consider that if a variable is biological then its mechanisms are sexed and not gendered (see the “Course 3: Sex and Gender in the Analysis of Secondary Data from Human Participants” of “Canadian Institutes of Health Research” website [16]). Yet social factors, like gender, can be “embodied” during life course [17, 18] and therefore have effects on biological processes. So, it is important to identify what, in biological and health differences between men and women, is gender-related and therefore may be modifiable by implementing prevention measures targeted at the relevant determinants.

To answer these questions, epidemiologists need tools to capture the sex and gender phenomena and these tools must be compatible with the discipline's methods. The first issue might be the need to contain the complex concept of gender in one or several individual variables to be used in epidemiological analyses. But questioning the influence of sex and gender on health does not only require correct measures, but also analytical approaches aiming to capture the phenomena of sex and gender in their complexity and in isolation one from another.

Our aim here was to clarify concepts, measures, paths, effects, and analytical strategies useful to explore mechanisms of health difference between men and women. We first reviewed concepts to clarify their definitions and limitations for their translation into usable measures in Epidemiology. We then conducted methodological research using a causal framework to propose a methodologically appropriate strategy for measuring gender effects in health, based on mediation analysis. Finally, we propose an alternative strategy based on the estimation of the interaction effect between sex and social environment.

## Gender, from a population-level concept to an individual variable

### How gender can be conceptualized in epidemiology

Gender is a *differential social construct*: it corresponds to the fact that norms of different kinds (behaviors, activities, experiences) are differently prescribed to individuals according to their attributed-at-birth sex. The gender system divides humanity according to the typology known as "sex" and associates values, objects and properties of different kinds with each of these categories. For example, physical strength, the color blue and mathematics might be considered as masculine objects, in a given place and time. This is consistent with a sociological approach which refers to gender as a process of social division: "*gender is the system of hierarchical division of humanity into two unequal halves*" [19]. Through socialization, we suggest that the process of division will be realized through individuals by a sex-differentiated normative pressure. This hypothesis follows Durkheim’s definition of social fact: "*these types of conduct or thoughts are external to the individual but, they possess an imperative and coercive power by virtue of which they are imposed on them, whether they like it or not*" [20]. This normative pressure will, in our example, encourage men to develop their strength, to wear blue clothes, to love mathematics. This differential normative pressure, based on attributed sex, creates a systematic and systemic difference between men and women in society, observed at population level. Indeed, even if individuals do not adopt all valued attributes of their sex, a difference will be observed in the distribution of these attributes among individuals categorized by sex at the population level.

### Translation into an individual measure

Gender is socially performed, through a systemic normative pressure, and observed as a distributional gap at the population level. However, in epidemiology, variables are typically defined at the individual level to be linked to other variables like health outcome. We observed three ways of measuring gender in epidemiology: gender identity, gender personality and gender diagnosis.

- Gender is often associated with *gender identity*, measured by self-reporting from an individual. This dimension of gender can be defined as how an individual sees themselves on the continuum of socially prescribed femininity or masculinity or outside this continuum [2]. It is highly dependent upon social prescriptions and other social determinants (e.g., capacity for self-determination) and is not necessarily related to what the individual *performs* through their behaviors, activities, etc.
- We also find gender personality scores such as: Bem-Sex-role-inventory [21], Conformity to Masculine Norms Inventory [22]. The measurement of these scores needs to be planned beforehand. Moreover, these scores are based on stereotypes and are not sensitive to the context of generation, culture, age, class, race, etc.
- Measures of "Gender diagnosis" kind [23] are composite individual indicators which can be constructed from data, based on the presence or absence of several gendered dimensions, defined from the sex-differential distribution of these dimensions in the population. This indicator corresponds to a measure of the level at which an individual complies with a set of elements constituting femininity or masculinity in a given population, place and time, i.e., as a kind of probability of being "predicted male" from dimensions associated with masculinity, or "predicted female" from dimensions associated with femininity. This means considering an individual as being more or less masculine because they have a greater or fewer number of masculine characteristics, considered as such because these characteristics are more frequent or of a higher value among men within the population studied. This is a common measurement method of gender in gender-sensitive studies [24, 25].

### Strength and limitations of gender-diagnosis measures

“Gender-diagnosis” measures are consistent with the way gender can be conceptualized at the individual level in epidemiology as the result of normative pressure resulting in a differential probability of having this or that characteristic according to birth-sex. This method can be used in secondary data analysis and also takes into account the context by being defined for a given population, so based on the specific norms of that population.

It has some limitations, however, which require precautions. Firstly, we will obviously never be able to capture the totality of such a diffuse and complex phenomenon in one or several variables. Secondly, as this phenomenon is not necessarily consistent at the individual level, we will lose information by reducing it into a score. For example, a man may have a job considered to be feminine, such as caregiving or midwifery, but display characteristics considered to be masculine in his family environment (measured from the burden of domestic tasks for example). To take this into account, several variables related to the different ways of characterizing groups and contexts could also be defined, depending on available data and constraints of the research question, for example "occupational gender" and "domestic gender", or, more often, "gender role" and "gender relationship" [5]. But we inevitably lose some of the nuance.

Thirdly, the presence of one or more gendered dimensions in an individual is not necessarily due to gender pressure alone. For example, if, in a given group, smoking is much more common among men than among women, this behavior will be considered as masculine. But the fact that an individual smokes is not determined solely by this mechanism. It is not only a marker of a person’s gender, but also a marker of other social determinants such as socio-economic position, social network, etc. If we observe an effect of this gender score on an outcome in a sex group, it is therefore difficult to interpret whether this effect is really due to the gender phenomenon we are trying to capture or to other factors determining the score.

We cannot therefore consider a gendered dimension, as a ‘pure and perfect’ proxy of individual gender. This limitation should be kept in mind when using gendered variables as gender markers. Despite its limitations, this “gender diagnosis method” remains a pragmatic tool.

## It is not just about sex and gender, it is about mechanisms

### Visualize links and context, using directed acyclic graphs

Even if the concepts of sex and gender have been well defined, differentiated and measured, with all the limitations mentioned, this is still not sufficient to isolate and analyze the effects of sex and gender on a health outcome, denoted *Y*. Mainly because individual gender, defined as the result of gender pressure on an individual, is strongly associated with sex: if a newborn baby is defined as male, he will be socialized as a boy, whereas if defined as female, she will be socialized as a girl. The gendered characteristics that each child will have, even if modulated by other social and individual factors, thus strongly depend on their sex, which is a "parent” (direct cause) of these characteristics, in a causal-framework sense. Therefore, an association between an individual score of gender and an outcome Y cannot be interpreted as proof that gender pressure explains part of Y, because sex is a confounder in this association. The reverse interpretation would be equally flawed: we cannot conclude from an association between birth-sex and an outcome Y that biological mechanisms only explain this association and not the gender pressure, because the effect of sex on Y can be mediated by (= can pass through) gender.

It is therefore insufficient to simply avoid confusing sex with gender concepts and the variables that measure them, we also have to avoid confusing the mechanisms that relate one to the other. To grasp these issues, we propose to use causal-approach tools to clearly identify the mechanisms of interest and, on this basis, define our analysis strategy: directed acyclic graphs (DAG) and counterfactual notations [26, 27]. The principle is to visually represent all the variables of interest (the "nodes"), measured or not, and all the possible causal links between these variables (the "arrows"). This tool allows us (1) to be transparent about the a priori hypotheses regarding the underlying causal structure; (2) to precisely define the effect to be estimated in order to meet the objectives (which can be expressed using counterfactual notations); (3) to build the appropriate model to identify and estimate this effect, taking into account the context and thus avoid the main methodological biases like not adjusting on a confounder or adjusting on a mediator, etc. [27].

**Fig. 1:**
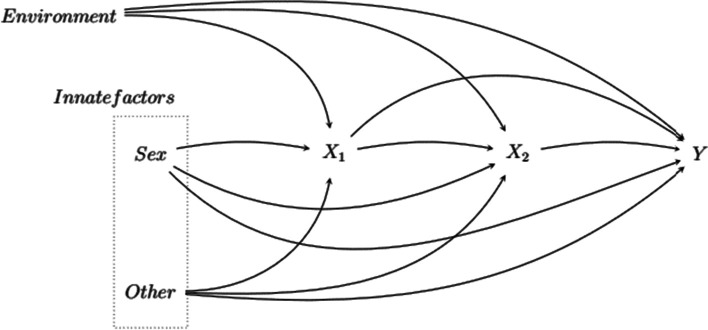
General graph of causal links

Figure 1 is an example of DAG, allowing us to visualize the sequence of causes and therefore the whole causal structure. This graph represents a very general scenario of a sequence of two exposures *X*_1_ and *X*_2_ that cause an outcome *Y*, each node representing a variable or set of variables. Fundamental and independent determinants of *X*_1_, *X*_2_ and *Y* are innate factors, including sex, and environmental factors.

### What are "sex effects" and "gender effects"?

**Fig. 2:**
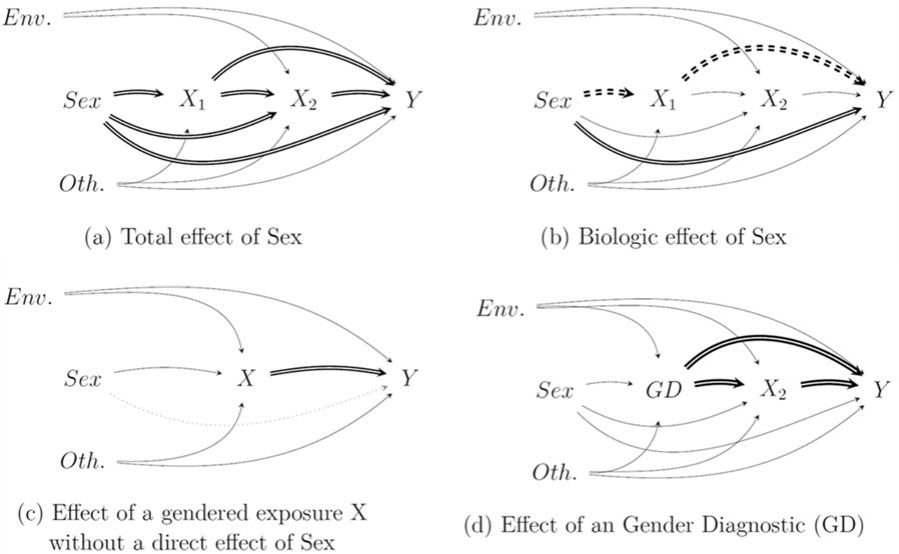
Effects of sex and gender: Total effect of Sex (**a**), Biologic effect of Sex (**b**), Effect of a gendered variable (**c**) and Effect of a gender variable (**d**)

Strictly speaking, the “effect of sex” on *Y* corresponds to all directed paths that begin at the *Sex* node and end at *Y* (double arrows in Fig. 2a). However, it is sometimes implicitly suggested that when we talk about the “effect of sex”, we are only talking about biological mechanisms and that we are therefore only referring to paths that would not pass through social factors. In fact, this “biological effect of sex” would be the direct effect (double continuous arrow in Fig. 2b) and the indirect effects which pass through exposures not linked to the environment (double dashed arrows, with the hypothesis of independence between *Env* and *X*_*1*_), and assuming that no mediators with social–environmental determinant have been omitted.

When we observe a result where there is an association between Sex and an outcome *Y*, this finding corresponds to the total effect of *Sex* on *Y* (Fig. 2a). Again, we cannot know if this total effect is explained by biological or social mechanisms, even if we have well defined the *Sex* variable as a biological phenomenon. It is therefore important to determine if this is really the effect of interest. By using these graphic representations, we can also highlight the complexity of isolating the biological effect of sex, which would require us to first make the strong assumption of an independence between the environment and all the intermediate factors (as for *X*_*1*_ in our example), and second to "block" all other paths to identify the direct effect.

We can also focus on gendered exposure(s). For example, if the probability of playing football *X* is different according to birth-sex and to the place of residence, we will say that this activity is socially determined and gendered. We would want to identify the risk factors for a rupture of the anterior cruciate ligament *Y*, assuming that there is no direct effect of sex on the probability of this pathology occurring but an effect of playing football *X* (see Fig. 2c). Since playing football is a risk factor for the disease and a gendered activity, we would find here a “sex effect”, i.e., statistical association and even a causal pathway (mediated by football *X*) between the variable *Sex* and *Y*. In this example, *X* is a gendered activity, but we could have used another gendered dimension, gender identity, a set of gendered variables or a gender diagnosis, as described above and as represented in Fig. 2d. This figure allows us to visualize the potential confounding effect of sex and environment when we look at the effect of this kind of gender marker on *Y*. If we wanted to identify and measure the specific effect of individual gender, we would have to make sure that we could control all these confounders. These examples demonstrate that it is necessary to ensure that the assumptions regarding mechanisms and pathways of interest are clearly defined a priori.

### Exploring mechanisms with mediation analysis

When we want to understand the health effects of sex and gender, i.e., describe them, distinguish them and explore their mechanisms, different questions can be addressed that do not involve the same analytical strategies. If the question is: “Are the differences in health observed between men and women explained, at least partially, by social mechanisms?”, then our focus will be on the pathways operating through social dimensions of a *Sex → Y* effect, i.e., in the *socially mediated indirect effect of sex*. If the question is “Does a gendered dimension(s), like a gender diagnosis, have an effect on health?”, then our focus will be on the total effect of a gendered exposure, as described in Fig. 2c. It is therefore important to distinguish, name, and define the multiple pathways that link sex and gender to the outcome.

Based on the causal framework, mediation analysis strategies [28, 29] have been defined in order to estimate mediated effects. These strategies could allow us to answer questions such as "how much of the sex-difference on that outcome is explained by gendered behaviors?” for example. Based on these methods and on the counterfactual formulation ("if the situation had not been as it is"), we propose a typology of several effects of interest in Table 1, with corresponding examples of counterfactual formulation. We will denote *Y*_*S*=*s*_ or *Y*_*S*=*s,*_ _*E*=*e*_ the potential outcome had a subject been exposed, respectively, to the counterfactual interventions *S* = *s* or {*S* = *s* and *E* = *e*}. In this table, the gender variable is described as a binary variable *G* = *{f;m}* in order to simplify the presentation rather than for a conceptual reason.

**Table 1:** Typology of sex S and gender G effects on a health outcome Y

Name	Definitions	Examples of counterfactual formulation
Total effect of sex (TES)	The difference in the value of *Y* had the whole population been born female “*S* = *f*” versus the whole population been born male “*S* = *m*” **Corresponds to the total effect of being born male (versus being born female) on Y value, whatever the mechanisms explaining these differences**	$E\left({Y}_{S=m}\right)-E\left({Y}_{S=f}\right)$
Socially mediated indirect effect of sex (SMIES)	The difference in the value of *Y,* had the sex been set to a constant level (for example “*S* = *m*”), and gender been change from *G*_*f*_ to *G*_*m*_ in the whole population **Corresponds to the indirect effect of sex which is *explained/ mediated* by social mechanisms *G***	$E\left({Y}_{S=m;G={G}_{m}}\right)-E\left({Y}_{S=m;G={G}_{f}}\right)$
Direct or residual effect of sex (RES)	The difference in the value of *Y* had the sex been changed from female “S = f” to male “S = m” in the whole population, while the gender variable been set constant to ${G}_{f}$ **Corresponds to the direct effect of sex which does not pass through *G***	$E\left({Y}_{S=m;G={G}_{f}}\right)-E\left({Y}_{S=f;G={G}_{f}}\right)$
Sex-controlled gender effect (SCGE)	The difference in the value of *Y* had the whole population been gendered in some way “G = f versus the other way “G = m”. Correspond to the total effect G → Y. In this case, the sex and the environment are confounding factors, it will therefore be necessary to adjust for them **Corresponds to the effect on G of Y “whatever the sex”**	$E\left({Y}_{G=f}\right)-E\left({Y}_{G=m}\right)$

With: S for Sex, G for Gender, Y for outcome

Ideally in this typology, *G* should represent "being / acting / living / etc. as a man" (or "as a woman"), i.e., everything that socially makes a man (or a woman) in a given time, place and population. In this case, the direct effect, RES (what does not pass through *G*), would correspond to the non-socially mediated or the biological effect of sex in these time, place and population. But, as we said before, gender is so diffuse that it is impossible to think that we can capture all its dimensions in one or a few variables. With this analytical strategy, at most we can: (1) verify the hypothesis that social pathways (SMIES) explain, at least in part, a sex effect (TES), and (2) have an order of magnitude of the biological effect of sex on a phenomenon *Y* depending on the conceptual extensivity of G. But a *RES* can never be said to be the pure biological effect of sex, even if we have considered many gendered dimensions in G.

## An alternative analytical strategy: gender as a sex–environment interaction

### Gender as a sex–environment interaction: conceptual arguments

Rather than using individual variables as proxies for a population-level phenomenon, gender could also be directly examined as the mechanism. This mechanism can be considered as an interaction between sex and social environment. Indeed, we said above that a dimension is gendered when it is a descendant of both sex and social environment, but there are cases where a dimension is a descendant of both sex and social environment and is not gendered. For example, let us imagine that the head circumference of newborns is on average different depending on sex at birth. In a given society, pregnant women of one caste eat differently from others and this diet has an effect on the head circumference of newborns. In this society, however, the sex of the child before birth is not known. In this case, the newborn’s cranial perimeter is a descendant of sex and the environment, but it is not gendered. It would be if the sex of their unborn child was known and if the pregnant women also ate differently according to this knowledge. So, the dimension is said to be gendered not only when it differs according to sex and to the social environment, but also when the environmental cause varies according to sex. It is the definition of an interaction phenomenon. This is why we can refer to gender as a "differential distributor" of social exposures. In terms of social and biological explanations, a sex-difference that exists whatever the social group and the culture is likely to be biological, but if its effect varies greatly according to social classes or cultures, it may be mediated by social mechanisms. This echoes anthropologist Margaret Mead’s conclusions that temperaments (gentleness, violence, etc.) attributed to men or women did not stem from biological sex but were socially constructed because they varied from one society to another [30].

### Gender as a sex–environment interaction: strategic implications

We can start from this definition to define a strategy that will consider gender as an interaction between sex and the social environment. Counterfactual formulations of interaction effects have also been proposed in the causal framework, as well as a methodology to choose the scale (additive or multiplicative), to estimate these effects and to present results [31]. We denoted the social environment *E*, with *E* = 0 the social-environment group of reference (the less gendered group ideally, as a non-gendered environment generally does not exist) and *E* = 1 the other group. We denoted S the attributed-at-birth sex. We want to distinguish the effect of sex, which would occur in the reference group; the effect of the environment, not related to a sex effect; and the effect of gender, as a sex-differentiated effect of the environment, or a socially varying effect of sex (equivalent formulation). The way of identifying effects is totally different from what we have proposed above, so we named them differently (Table 2).

**Table 2:** Typology of sex S, social environment E and gender G effects on a health outcome Y

Name	Definitions	Counterfactual formulation
Without-gender total effect of sex (WOGTES)	The effect of sex that would be found in a social environment with the minimal gender phenomena (*E* = 0) In practice, this effect more reasonably corresponds to a combined effect of sex and a minimal gender pressure, close to what RES could also mean	$E\left({Y}_{S=1;E=0}\right)-E\left({Y}_{S=0;E=0}\right)$
Without-gender total effect of environment (WOGTEE)	The proper effect of the environment not due to the gender phenomena (ideally), i.e., which not vary with sex	$E\left({Y}_{S=0;E=1}\right)-E\left({Y}_{S=0;E=0}\right)$
Total effect of gender (TEG)	The difference of the total effect of sex between two social group = the difference of the total effect of the social environment between two sex group (a kind of avoidable effect of the social environment) These two formulations are conceptually and counterfactually equivalent and corresponds to the writing of an additive interaction between sex and environment	$$\begin{array}{c}\left[E\left({Y}_{S=1;E=1}\right)-E\left({Y}_{S=0;E=1}\right)\right]\\ -\\ \left[E\left({Y}_{S=1;E=0}\right)-E\left({Y}_{S=0;E=0}\right)\right]\end{array}$$

With: s for sex, *e* for social environment, g for gender defined as an e*s interaction, y for outcome

As with the previous strategy, some limitations are found. Firstly, we simply shift the problem of measuring a complex concept with one or several variables from the realm of gender to that of the social environment. Secondly, even if the WOGTES was large and the TEG null, cannot to conclude that Y are not at all influence by gender, because the measured effect would depend on the social environment variable chosen, and because a perfect non-gendered group of reference is not realistic (except some special cases like sex-blind in utero environment). Thirdly, an important condition for the successful use of this strategy would be to have a very socially heterogeneous population in order to estimate the variability of the gender effect across social groups by this TEG. On the other hand, the interest of this strategy is to be more compatible with the population-level nature of gender, considered as a sex–environment interaction or taking into account sex as a modifier of the effect of the environment. This approach makes it possible not to define a measure of gender which, even if defined in a study in a contextual way and with all precautions, always runs the risk of being generalized and essentialized afterwards.

## Discussion

### Choosing and conducting the strategy

We identified two methodological strategies to explore the mechanisms of health differences between men and women: (a) defining and measuring gender as a social mediator of sex or (b) defining and measuring gender as a sex–environment interaction. Here, we provide different arguments to help decide between them and recommendations to conduct them.

1. A fundamental criterion to select is the type of study population: strategy (a) is more appropriate if socio-cultural characteristics of the population are rather homogeneous; and strategy (b) is more appropriate if socio-cultural characteristics of the population are heterogeneous.
2. According to the research question, specify the chosen strategy and, with the help of tools such as directed acyclic graphs, define the specific effect to be estimated and the necessary methodological precautions [27].
3. Define required variables:

- If strategy (a) is chosen, use available data and mechanistic assumptions to define variables for defining a gender score, based on the distribution of these variables according to sex in the study population (gender diagnosis), or keep the social characteristics of interest separated if methodologically possible.
- If strategy (b) is chosen, define a summary variable of the social environment in the sample and globally assess the variability of differences between men and women according to this variable in the study population (e.g., income gap, employment access gap, age of first child, etc.). Define a reference group.

(4) Discuss the limitations of the chosen method and interpret the results with these precautions.

- If strategy (a) is chosen, evaluate and discuss the share of the gender phenomenon captured by the individual gender variable(s) and the share of non-gender-related phenomena also possibly captured (variability of the measurement depending on other social characteristics for example).
- If strategy (b) is chosen, evaluate and discuss the share of the gender phenomenon captured: is the population sufficiently heterogeneous? Has it been possible to characterize the different socio-cultural groups with the "social environment" variable? How and how much are the various socio-cultural groups differently gendered?

(5) Gender effects could then be qualitatively explored through: (i) the outcomes: for what health outcome do we observe a social mediation or a sex–environment interaction? (ii) the groups linked to the social environment: between which social groups do we observe or do not observe a difference in the sex effect? How and how much are these groups visibly gendered? (iii) the mediators: through what mediators does the gender effect or the interaction effect pass?

### Limitations

These strategies must be understood within the defined perspective of understanding the mechanisms of differences between men and women in health. Studies that focus specifically on intersex, transgender, transsexual populations would require other approaches that are not described here. These strategies are also suited to a comprehensive and exploratory approach to the issue only: it seeks to explore the nature (biological or social) of observed differences between men and women when it does not seem trivial. The construction of a gender score seeks to capture a latent phenomenon, but does not necessarily imply that the variables used for this score are the risk factors for the health outcome. No a priori assumptions are therefore made on the type of specific exposures involved, which could be modified from a public health perspective. This objective could come in a second step, once the involvement of social mechanisms would have been identified.

Since the gender phenomenon is, as we have reiterated, contextual, the score constructed in a study on a specific population cannot be directly transposed to another population. The use of this kind of score can lead to the conclusion that a difference in health between men and women is, at least partly, explained by social mechanisms. However, the estimated size of the effect of these mechanisms could not be generalized to other populations, since the ways in which gender pressure performed in these other populations are likely to differ and therefore to influence health outcomes differently. The alternative approach, based on the study of sex–environment interaction, might more easily avoid the risk of essentializing differences, since it is precisely based on the variability of the involved processes. It may also make it possible to capture population phenomena by characterizing groups from the level of gender inequalities observed within them. These phenomena are otherwise difficult to capture with epidemiological methods based on individual-level variables, while it is a central aspect of understanding the gender process, based not only on the sex-differentiated norms prescription, but also on their interrelated hierarchical relations.

We argue that a causal analysis framework can guide us to refine our objectives, assumptions and conduct more rigorous analyses. It is from these methods that our approach has been built. However, this method has some drawbacks, including the need to define, for each factor, its "counterfactual". Firstly, this can give the impression that a binary categorization is unavoidable or that we are reinforcing it: if I was not born a female, it is because I was born a male; if I am not socialized as a woman, it is because I am socialized as a man. In practice, this may correspond to the way in which, in a gendered society based on a bipolarization of classifications, people are actually exposed or not to a kind of socialization. But this binarity must not be essentialized. A continuous masculinity (or femininity) score could also be constructed, its counterfactual formulation would be: "being socialized in a very masculine way" versus "being socialized in a less masculine way", without considering feminine socialization as the exact symmetry of this measure. Secondly and most importantly, definition of counterfactuals is constrained by reality since models are estimated from what is observed. So, it will usually not be possible to compare sex health differences observed in a given population with sex health differences “that would be observed in a population that would not know the gender phenomenon”, when only this comparison would really meet our objective. Similarly, the effect of a sex-independent gendered socialization will not be optimally measured, because, in our societies, moving from a female-socialization to a male-socialization for a female individual will never be equivalent to moving from a female-socialization to a male-socialization for a male individual. Perfect counterfactuals do not exist where gender is concerned.

### Conclusion

Both analytical strategies have limitations relative to the impossibility of reducing a complex concept to a single or a few measures, and of capturing the entire effect of the phenomenon. However, these approaches, supported by causal framework, could lead to more accurate analyses of the mechanisms underlying health differences between men and women, and may ultimately limit the gender bias encountered in epidemiological and clinical research studies.

## Perspectives and significance

In this article, we clarified concepts and measures of sex and gender. We argue that directed acyclic graph, used in causal framework to clearly formulate a priori assumption about links and direction of links, clarifies what is measured and reduces methodological bias. We proposed two analytical strategies depending on how we measure gender: as a social mediator of sex effect or as a sex–environment interaction. We provide different arguments to help decide between the first or the second strategy. Both analytical strategies have limitations relative to the impossibility of reducing a complex concept to a single or a few measures, and of capturing the entire effect of the phenomenon. Despite these limitations, these approaches, supported by causal framework, could lead to improve the coherence between complex SHS concepts and the analytical approach in epidemiology in the exploration of mechanisms underlying health differences between men and women.

## Data Availability

Not applicable, no data used.

## Footnotes

1 Defined as a discipline which manipulates quantitative or categorical variables defined at individual level to measure statistical associations, causal relations and mediation paths at populational level, with the objective of understanding etiological mechanisms behind health or disease.
